# Numerical Modeling of a New Type of Prosthetic Restoration for Non-Carious Cervical Lesions

**DOI:** 10.3390/ma15155102

**Published:** 2022-07-22

**Authors:** Anna A. Kamenskikh, Lyaysan Sakhabutdinova, Nataliya Astashina, Artem Petrachev, Yuriy Nosov

**Affiliations:** 1 Department of Computational Mathematics, Mechanics and Biomechanics, Perm National Research Polytechnic University, 614990 Perm, Russia; lyaysans@list.ru (L.S.); ura.4132@yandex.ru (Y.N.); 2 Department of Orthopedic Dentistry, Perm State Medical University Named after Academician E.A. Wagner, 26 Petropavlovskaya St., 614990 Perm, Russia; caddis@mail.ru (N.A.); artem@petrachev.ru (A.P.)

**Keywords:** tooth, NCCL, contact, modeling, finite element method (FEM), biomaterials, strain

## Abstract

The paper considers a new technology for the treatment of non-carious cervical lesions (NCCLs). The three parameterized numerical models of teeth are constructed: without defect, with a V-shaped defect, and after treatment. A new treatment for NCCL has been proposed. Tooth tissues near the NCCLs are subject to degradation. The main idea of the technology is to increase the cavity for the restoration of NCCLs with removal of the affected tissues. The new treatment method also allows the creation of a playground for attaching the gingival margin. The impact of three biomaterials as restorations is studied: CEREC Blocs; Herculite XRV; and Charisma. The models are deformed by a vertical load from the antagonist tooth from 100 to 1000 N. The tooth-inlay system is considered, taking into account the contact interaction. Qualitative patterns of tooth deformation before and after restoration were established for three variants of the inlay material.

## 1. Introduction

### 1.1. Research Objectives

The object of the study is new prosthetic inlays in non-carious cervical lesions (NCCLs). They suggest the expansion of the cavity of the NCCLs.

Research objectives:

- the creation of parametrized models of teeth with and without the NCCL;
- the creation of parametrized models with the restoration of the NCCL in the form of a new prosthetic inlay;
- the implementation of a series of numerical experiments on strain of the tooth before and after restoration;
- the analysis of the impact of prosthetic inlay materials on strained teeth.

The development of a new method for replacing NCCL and a preliminary assessment of the effect of restoration materials on the tooth-inlay system deformation are the main goals of this study. This method can increase the restorations’ service life and their aesthetics. An NCCL is often combined with gingival margin recession. The new treatment method allows the creation of a playground for attaching the gingival margin. The evaluation of the restoration materials’ performance is also an important factor.

The work includes only computational experiments at this stage. All studies are in silico. The analysis of therapy parameters and the preliminary selection of materials occur on numerical models.

### 1.2. Problem Context

Computer modeling and the accumulated experience of describing the behavior of various materials allow them to be used in the field of medical research. Today there are many examples of successful applications of numerical models in dentistry [1,2]. Research of the finite element method (FEM) allows us to expand our understanding of the causes of the occurrence and development of mechanical damage to teeth. Analysis of NCCLs in terms of mechanical behavior seems interesting and promising [3,4].

NCCLs are a fairly common dentition disease in the world [5,6,7]. This is a non-carious disease of the teeth with tissue degradation near the cervical area. There are four types of NCCLs according to the nature of development in the tooth tissues, as well as two types according to the geometry of the “wedge” section (V and U shaped) [8,9]. It is scientifically substantiated that the shape of the NCCLs affects the load distribution in the tooth. In the area of development of a V-shaped NCCL, the stress concentration is four times higher [9]. Tissue degradation in the defect zone develops without treatment. It has a significant impact on the patient’s life quality. Such NCCLs cause a number of inconveniences to the patient: violation of the tooth aesthetics, pain, overload of healthy teeth, etc.

There are many works aimed at studying the causes of the occurrence and development of NCCLs. The hard tooth tissue properties of NCCLs affected are being investigated [10,11,12,13,14]. The biomechanical reasons for the occurrence of abfraction are analyzed [12,15,16,17]; Occlusal loads and resulting stress-strain states of teeth are being studied [16,18,19,20]. It is found that the enamel strength decreases in the direction from the outer surface to the dentine-enamel junction [21]. The axial load is 30% more strongly distributed in the enamel. The response to axial load is 30% higher in the enamel than in the rest of the tooth tissues. Multiple multidirectional loads cause reactions that are five times higher than the reactions to the axial load of the same level [17,18]. At the same time, the enamel-cement border and the cervical part of the vestibular surface experience the maximum load, mainly in the incisors and premolars. Abfraction is considered the main cause of developing tooth NCCLs [12,22,23]. However, Grippo J.O. and Masai J.V. [24] found that the stresses occurring in the cervical region on the vestibular surface are similar to those on the oral one, where NCCLs are extremely rare. The combination of acids and internal stresses on tooth enamel is the reason for this effect according to scientists [24,25]. A relationship is also established between parafunctions, such as bruxism, and the occurrence of tooth NCCLs [26,27].

The loss of restoration of NCCL is a serious problem that dentists face daily. This is due to many factors, for example, high loads on the tooth crown part [11,12,28]. Part of modern research is aimed at analyzing the effect of the material on the strain of the tooth-restoration system [29,30,31]. Other authors consider the influence of different mechanisms of inelastic strain on the restorations’ performance [32,33]. The modification of the restorations’ geometric configuration and new treatment technologies is another research area [34,35]. Science-intensive approaches in dentistry have made it possible to study the processes occurring in the tooth tissues [10,15,19,36,37,38,39]. The influence of occlusal and parafunctional loads is researched. An analysis of change in the properties of hard tooth tissues is performed. The pattern of the stress distribution in healthy and affected teeth is investigated. However, to date, the information obtained on the etiology and pathogenesis of abfractions is still insufficient to provide quality care to patients with NCCLs.

This problem requires new effective solutions. The modern level of providing highly qualified medical care makes high demands in the treatment of dental system pathologies. The methods of biomechanical modeling and mathematical analysis acquire a special role in the planning stage of dental treatment [11]. FEM is used for the modeling and analysis of complex systems, including biomechanical ones [9,18,32,33,34,35]. The numerical model will allow planning options for the formation of a cavity for optimal long-term restoration. Changing the defect geometry during preparation can change or transfer the load vector to stronger areas. The prediction of the impact of loading for various restoration options will help to assess the possible effectiveness of orthopedic treatment. A reasonable choice of treatment tactics will prevent or reduce the rate of progression of the disease.

### 1.3. Problem Description

An NCCL is a fairly common tooth lesion. The disease causes increased tooth sensitivity and violation of dentition aesthetics. NCCLs are located near the cervical area. The tooth perceives the worst loads from the antagonist’s. Further development of the defect is possible. With the disease progression, movement of the gingival margin in the apical direction is possible, which leads to developing the gingival recession.

An NCCL and its development cause a change in the tissues surrounding the lesion [40]. According to research, a local detachment of enamel from dentin was found in 30% of clinical cases. The separation causes a gap. The gap leads to the breaking off of the enamel area and the development of the defect in the future [25]. According to existing data, enamel changes are of a different nature. Focal enamel demineralization occurs in the areas around the defect [25]. Demineralization is aggravated when exposed to an acidic environment [41]. Various lesions are fixed in the enamel. The nature of the damage depends on the defect form and the main cause of its development. The damage can take the form of microscopic furrows, cracks, and craters [42,43]. Microdamages can lead to enamel chipping and defect expansion in the future [25,44]. The directions of the cracks do not have a definite dependence. Cracks propagate deep into the pulp chamber in the direction of the lesion, also in different directions from the defect [41,44]. Dentin changes occur in the NCCL zone and depend on the shape and depth of the defect [44,45]. Replacement dentin appears with deep damage and prevents the expansion of the cavity with gradual development NCCLs [25,46].

At the moment, the most widespread are direct restorations. However, they have a number of disadvantages:

- polymerization shrinkage;
- dependence on the manual skills of a particular specialist;
- short service life;
- pigmentation;
- the inability to ensure reconstruction of the periodontal attachment between the artificial material and the gum.

The necessity to create new technologies and methods for NCCL restoration has arisen. The new prosthetic inlays with an additional expansion of the wedge cavity are one of the solutions. The proposed method involves a significant amount of tissue preparation, both to expand the defect and to create zones of additional retention. It is important to note that mainly tooth tissues with accumulated macro- and micro-damages are removed.

Deformation of the tooth before and after restoration by a new method is considered in the work. Modeling is performed in the ANSYS Mechanical APDL application package (ANSYS Inc., Canonsburg, PA, USA). The tooth geometry is modeled as a first approximation and is more rounded. Crown geometry can be changed. In the first approximation, the crown geometry is not symmetrical and has different heights, i.e., an attempt is endeavored to bring the geometry of the crown closer to the individual case.

## 2. Materials and Methods

### 2.1. Model

**Figure 1:**
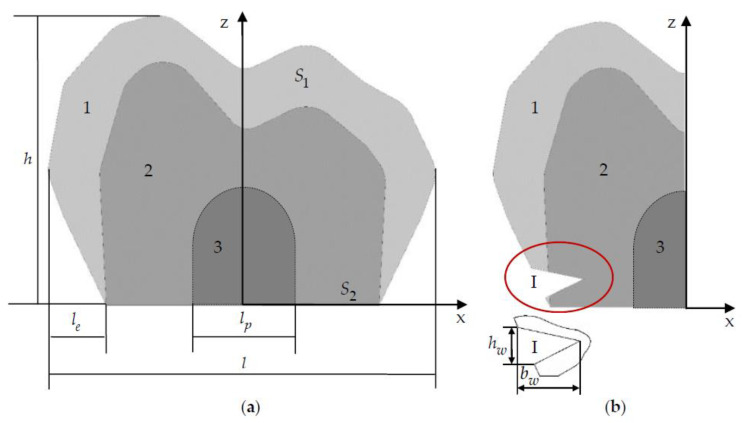
The central section of the tooth, taking into account (**a**) and without taking into account (**b**) an NCCL: 1 is enamel; 2 is dentine; 3 is pulp; I is defect.

The central section of the tooth, with and without taking into account the NCCL, is shown in Figure 1. The tooth model includes the volume of enamel (1) and dentine (2). The tooth pulp (3) is not modeled but is taken into account when parameterizing the NCCL. When deepening the NCCL in the tooth tissue, it is taken into account that it should not penetrate into the pulp.

The tooth geometry without taking into account the root system is often used in practice [47]. Such models make it possible to quickly obtain qualitative results on the unit deformation. The tooth models do not take into account the root system. This decision was made to qualitative assessment of tooth deformation in the first approximation.

Parameterization of the tooth geometry is performed according to its main parameters: height h, length l, and thickness of the enamel $l_{e}$. $l_{e}$ is the parameter of the enamel’s maximum possible thickness. The actual enamel thickness in the model can be more or less by 10–15%. Figure 1 shows the geometrical configuration of a tooth with h≈7.2, l≈9.44, and $l_{e}\approx 1.5$ mm. Overall dimensions of the tooth correspond to premolars and molars. Static boundary conditions are set on the tooth surface $S_{1}$ and kinematic ones on the surface $S_{2}$. The boundary conditions for all models are the same. The load varies from 100 to 1000 N. The NCCL is parameterized. Parameterization is based on the position and coordinates of the defect. As a result of modeling, an NCCL (I) $2l_{w}\times h_{w}\times b_{w}$ is obtained. Figure 1 shows the NCCL (I) with parameters 5.38×0.73×1.3 mm.

**Figure 2:**
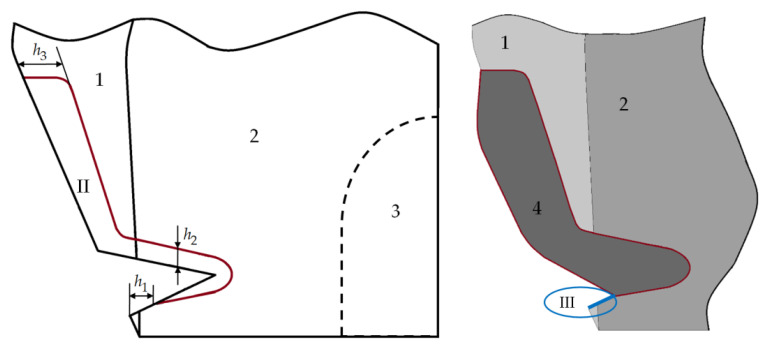
Modeling a cavity for a prosthetic inlay: 1 is enamel; 2 is dentine; 3 is pulp; 4 is inlay; II is cavity to fix the inlay; III is gingival fold.

A cavity is created in the tooth for a prosthetic inlay when creating a new type of restoration. Figure 2 shows the geometry of the cavity central section of the tooth model prosthetic inlay.

An original new method of treatment is proposed. The formed cavity to fix the inlay (II) includes the main part obtained by expanding the lesion, with an additional retention point in the form of a cavity passing to the proximal surface of the tooth, an additional platform at the top of the cavity for fixing the veneer part of the inlay, and a gingival fold (III) located more apically than the lesion.

Element (III) was introduced into the construct to recreate the dentogingival attachment. Often the NCCL is combined with a gingival margin recession. The reconstruction of the periodontal attachment between the artificial material and the gum is not possible.

The inlay cavity was parameterized using 3 parameters:

- $h_{1}$ is to create a gingival fold;
- $h_{2}$ is to create the main cavity;
- $h_{3}$ is to create an additional retention point with an additional platform at the top of the cavity for fixing the veneer part of the inlay.

**Figure 3:**
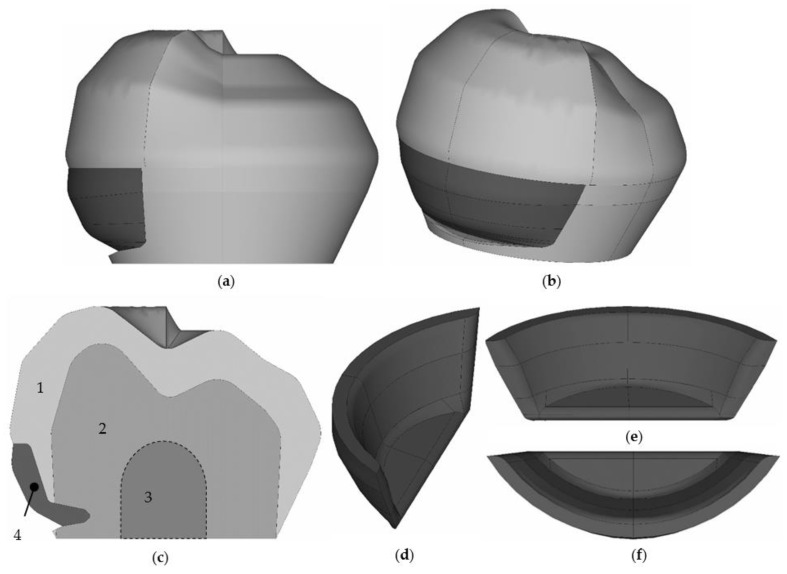
Tooth with a prosthetic inlay: (**a**,**b**) are isometry; (**c**) is the central section; (**d**) is inlay isometry; (**e**) is inside view inlay; (**f**) is top view inlay; 1 is enamel; 2 is dentine; 3 is pulp; 4 is inlay.

A geometric configuration of the inlay cavity is shown in Figure 3 for parameter values $h_{1}=0.3$, $h_{2}=0.2$, and $h_{3}=0.5$ mm. The central section of the cavity extends over the entire length of the NCCL. The inlay’s final appearance is formed with the geometry of the veneer part.

Figure 3 shows the tooth geometry with the NCCL restoration using a new prosthetic inlay, as well as the view of the inlay in the tooth cavity. Model 1 is enamel, 2 is dentin, and 4 is a new restoration of an NCCL using different materials. The pulp of the tooth (3) is not modeled but is taken into account when creating a cavity for a prosthetic inlay.

The inlay is quite streamlined on all sides. The inlay model is parameterized. The thickness, veneer part, and cavity area can change. The figure shows one of the options for the veneer part geometry of the inlay. Given inlay geometry will be used in a numerical experiment series.

The prosthetic inlay maintains the aesthetics of the dentition. The inlay restores the aesthetics of the tooth and increases the contact area of the tooth and the prosthetic structure.

The main limitations of the model at the moment:

- roots and gingiva are not taken into account in the model to save computational resources and a detailed study of the tooth-inlay contact zone;
- only the vertical load from the antagonist tooth in a wide range is considered;
- only the case of complete adhesion of the inlay and tooth is considered, although in reality sliding is possible;
- the elastic deformation behavior of materials is considered at this stage. It is planned to study the effect of heat shrinkage on the stress state of the biomechanical unit and refine the behavior model of the system materials in the future;
- the degradation of materials is not taken into account, as well as the formation of cracks due to the complexity of such mechanical models.

### 2.2. Mechanical Properties of the Mouthguard Components

The materials of the model are considered in elastic formulation. The elastic compression modulus (E) and Poisson’s ratio (v) of enamel and dentine are shown in Table 1. Properties of dentine and enamel are taken from reference literature.

The materials for the prosthetic inlay: CEREC Blocs (Sirona, Bensheim, Germany) is material 1; Herculite XRV (Kerr Corp, Orange, CA, USA) is material 2; Charisma (Heraeus Kulzer GmbH, Hanau, Germany) is material 3. The physical-mechanical properties of the inlay materials are presented in Table 2. The material properties for restoration are taken from reference literature [48,49].

The most promising material for creating an inlay is considered to be fine-structured feldspar ceramic blocks of industrial production CEREC (material 1). The material is used to make inlays, onlays, crowns, and veneers. The minimum values of the cavity parameters for the inlay are selected according to the restrictions imposed on the material when milling.

For comparison, inlays from two different composite materials are considered. Herculite XRV (material 2) is a versatile microhybrid composite material. Charisma (material 3) is a radiopaque glass-based composite material.

### 2.3. Numerical Finite Element (FE) Solution and Convergence

The simulation is implemented in the applied ANSYS Mechanical APDL engineering analysis package (ANSYS Inc., Canonsburg, PA, USA). Volume finite elements SOLID185 (four-node tetrahedra) with Lagrangian approximation and three degrees of freedom at each node are used. Contact gluing is modeled in the inlay-tooth interface zones, taking into account friction. The model eliminates the divergence of contact surfaces and appearance of sliding zones. The contact interaction is modeled using a contact pair of elements (CONTA173, TARGE170). The surface-surface contact is considered. The contact algorithm is the extended Lagrange method.

The finite element partition of the model is chosen within the assessment of the influence of the system discretization degree on the numerical solution of the problem. The minimum overall dimension of the finite element near the tooth-inlay contact area is 0.05 mm. When moving away from the contact zone, the size of the finite elements increases in a gradient. The maximum overall dimension of the final element reaches 0.15 mm.

## 3. Results

The crown part of the tooth is deformed together, i.e., there is no change in the crown geometry. The position and number of the points of load application from the antagonist tooth has little effect on the stress-strain state of the tooth-inlay system.

**Figure 4:**
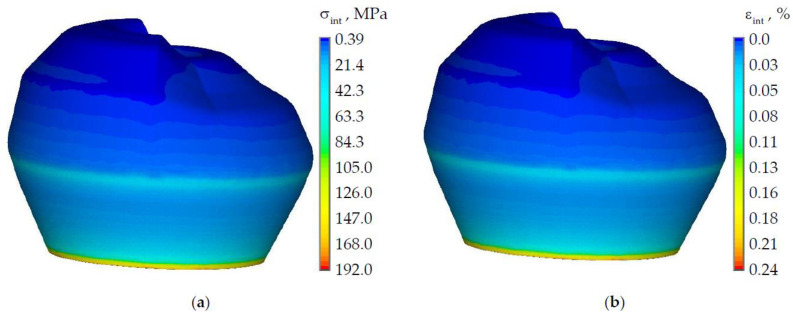
Stress and strain intensity of the tooth without NCCL: (**a**) is stress intensity; (**b**) is strain intensity.

The stress-strain state of a tooth without defect was considered in advance (Figure 4). The nature of the distribution of the intensity of stresses and strains is shown on the example of the load of 500 N from the antagonist tooth.

The maximum stress and strain intensity is observed in the zone of kinematic boundary conditions. The maximum intensity of stresses and strains is observed in the tooth enamel near the cervical area. The maximum stress level in dentine is 85% lower than in enamel and reaches 28.8 MPa. The maximum level of strains in dentine does not exceed 0.2%. The intensity of stresses and strains in the zone where the NCCL will be modeled reaches the level of approximately 70 MPa and 0.1%, respectively.

**Figure 5:**
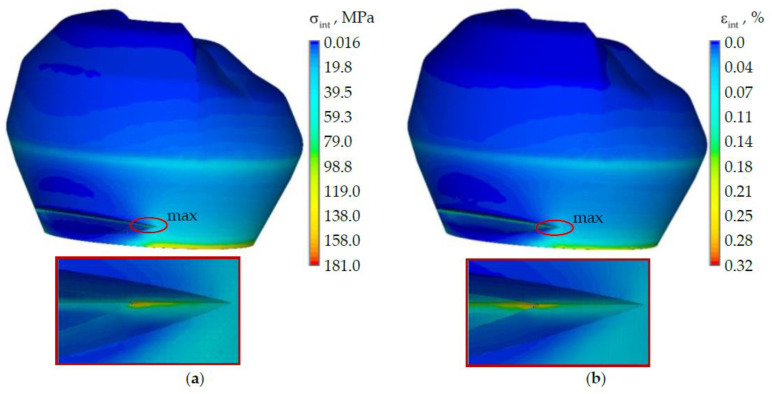
Stress and strain intensity of the tooth with NCCL: (**a**) is stress intensity; (**b**) is strain intensity.

The stress and strain intensity of the tooth with NCCL at a load of 500 N are shown in Figure 5.

The maximum level of stress and strain in the tooth model with NCCL is observed in the “wedge” zone. The maximum stress intensity is observed at the edge of the NCCL in the enamel and reaches 181 MPa. The maximum stress intensity is higher more than 2.5 times than in the tooth without NCCL. The maximum strain intensity is also observed in the NCCL in the dentine and reaches 0.32%, which is 1.6 times higher than in the model without defects.

**Figure 6:**
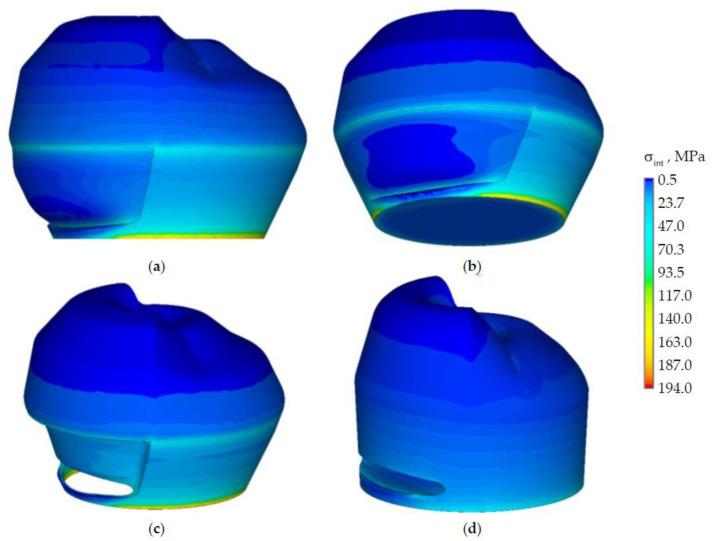
Stress intensity of the biomechanical assembly: (**a**,**b**) are tooth-inlay system; (**c**) is enamel; (**d**) is dentine.

**Figure 7:**
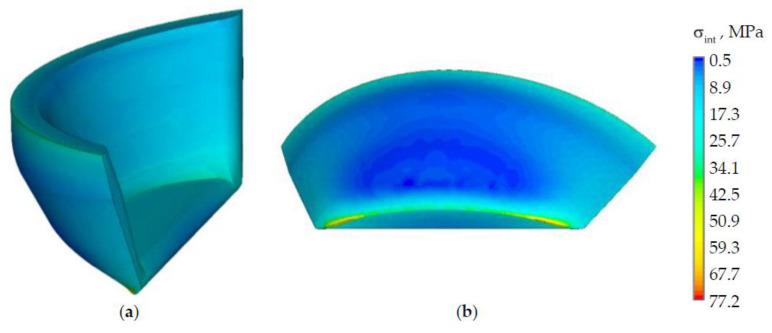
Stress intensity of the inlay: (**a**) is general view, (**b**) is outer side.

The next stage of the study is analyzing the effect of the prosthetic inlay in the NCCL by changing the “wedge” geometry. Figure 6 and Figure 7 show the stress intensity distribution in the biomechanical tooth-inlay system under the load of 500 N from the antagonist tooth. The qualitative view of the distribution of the deformation behavior parameters of the tooth-inlay system does not depend on the inlay material. The main difference between the solutions is in quantitative values. Stress and strain intensities are shown in the example of a model with an inlay from material 1.

The level of the stress intensity in the area of the prosthetic inlay is comparable to the stresses in the tooth without defects. The maximum stress intensity in the tooth-inlay system has shifted to the cervical area of the tooth.

The stress intensity in the inlay is 60.2% lower than in the tooth. At the lower boundary from the outside, local stress concentrators are observed at the level of 77 MPa. The distribution of stress fields in dentine corresponds to the loading conditions. The main stresses from the antagonist tooth action are realized in the enamel and in the inlay. The stress intensity on the outer surface inlay is lower than on the inner one.

**Figure 8:**
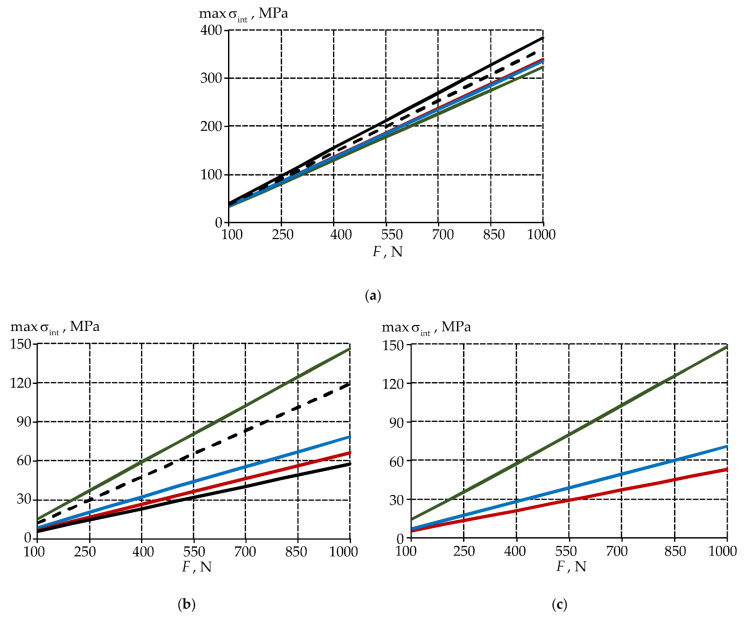
Dependence of the maximum values of the biomechanical assembly stress intensity on the load: (**a**) is enamel; (**b**) is dentine; (**c**) is inlay; black (solid line) is model without defect; black (dotted line) is model with defect; green is model with material 1 inlay, red is model with material 2 inlay, blue is model with material 3 inlay.

The dependences of the maximum values of stress intensities in the biomechanical system elements on the applied load value are shown in Figure 8.

The inlay material does not significantly affect the values of maximum stresses in the enamel. The more uniform distribution of the stress intensity in the biomechanical tooth-inlay system is observed by the use of material 1. A significant increase of stress intensity in the tooth dentin is observed in this case. The $\max\sigma_{\mathrm{int}}$ in dentine when inlay material 1 is 2.5 and 1.2 times higher than in the tooth model without and with an NCCL, respectively. A decrease $\max\sigma_{\mathrm{int}}$ in enamel and an increase in dentine were also observed when using prosthetic inlays from materials 2 and 3. A comparison of the stress level with models without and with NCCL is shown in Table 3.

A decrease in the stress intensity in the tooth enamel when using a restoration in the form of a prosthetic inlay by 12–16% can be noted. An increase in the maximum intensity of stresses in the dentine and the inlay near of the gingival fold is observed due to the contact gluing. The stress intensity in the model with a prosthetic inlay made of material 1 is more than two times lower on the main volume of materials. The influence of the geometry of the cavity for the inlay and veneer part on the deformation behavior of the biomechanical assembly must be studied.

**Figure 9:**
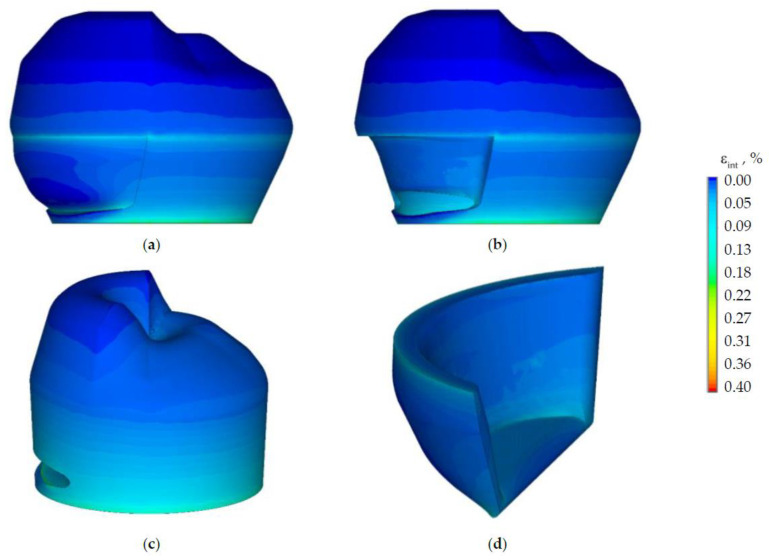
Strain: (**a**) is tooth-inlay system; (**b**) is enamel; (**c**) is dentine; (**d**) is inlay.

Let us consider the nature of the strain intensity distribution in the biomechanical tooth-inlay assembly (Figure 9).

The maximum strain intensity is observed in the dentine near the edge of the contact interaction zone with the prosthetic inlay. The strain intensity is lower by two or more times on the rest of the dentine volume. The $\max\varepsilon_{\mathrm{int}}$ in the enamel is observed near the cervical area of the tooth. The $\max\varepsilon_{\mathrm{int}}$ in the prosthetic inlay is located on the lower surface near the edge of the contact zone. This effect can be eliminated by: changing the geometry of the prosthetic inlay; the selection of material and refinement of the finite element model.

**Figure 10:**
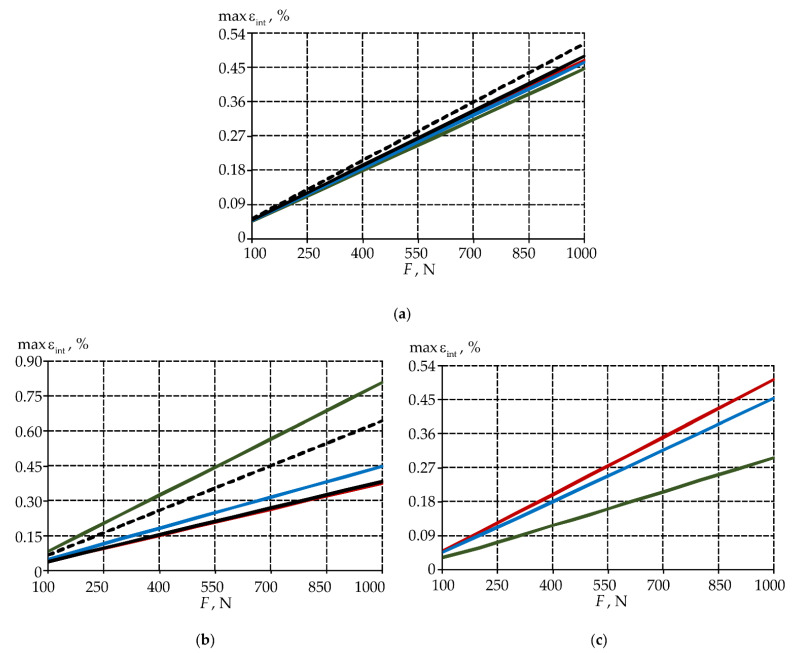
Dependence of the maximum values of the strain intensity of the biomechanical assembly on the load: (**a**) is enamel; (**b**) is dentine; (**c**) is inlay; black (solid line) is model without defect; black (dotted line) is model with defect; green is model with material 1 inlay, red is model with material 2 inlay, blue is model with material 3 inlay.

A dependence of the strain maximum intensity on the load level is shown in Figure 10.

The strain intensity in the enamel is lower in the models with a prosthetic inlay in the NCCL. The maximum influence on the nature of the distribution and the level of strain intensity is observed in the tooth dentine. The strain increase in the dentine and a shift of the maximum level $\varepsilon_{\mathrm{int}}$ to the “wedge” area in model with a defect can be noted. The $\max\varepsilon_{\mathrm{int}}$ dentine in the model with an NCCL is 1.7 times more than in the tooth without a defect.

The use of a new NCCL restoration in the form of a prosthetic inlay makes it possible to reduce the strain intensity in the dentine when using materials 2 and 3. An increase $\max\varepsilon_{\mathrm{int}}$ in dentine is observed in the model with an inlay made of material 1 by 2.1 times than in the tooth without defect. The maximum level zone $\varepsilon_{\mathrm{int}}$ is localized near the inlay-tooth contact border near the area of the gingival fold. The level of strain intensity is comparable to the strains of the tooth without defect on the main volume of dentine.

The minimum value $\max\varepsilon_{\mathrm{int}}$ is observed in the inlay from material 1. The maximum strain level of the inlay from materials 2 and 3 is comparable to the tooth enamel. This can adversely affect the service life of the prosthetic structure.

**Figure 11:**
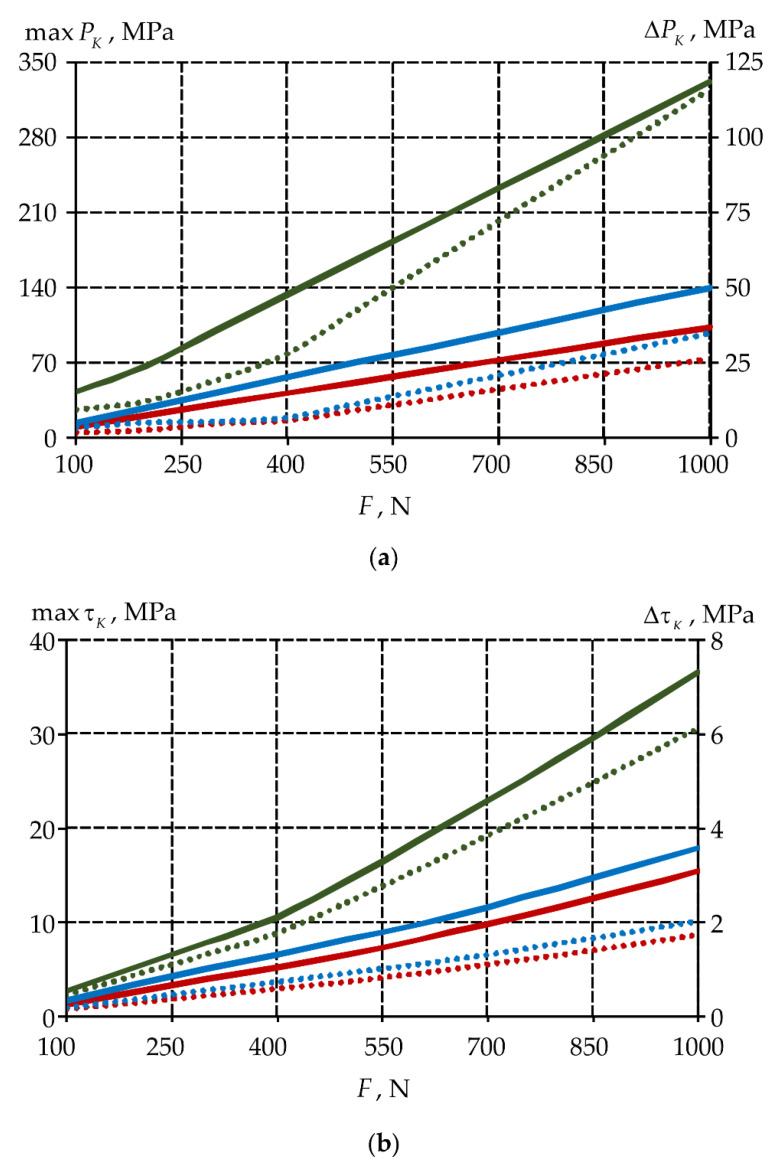
Dependence of contact parameters in the inlay on the load: (**a**) is contact pressure; (**b**) is tangential contact stress; solid lines is the maximum; points is the average; green is model with material 1 inlay, red is model with material 2 inlay, blue is model with material 3 inlay.

It is important to evaluate the dependence of the contact parameters because of the problem statement. The interface zone parameters are indicators of the strain behavior of the tooth-inlay system: contact pressure $P_{K}$ and contact tangential stress $\tau_{K}$. A dependence of the maximum (max) and average (Δ) levels of contact parameters on the tooth-inlay mating surface for three inlay materials are shown in Figure 11.

The maximum contact parameters are observed near the edge of the tooth-inlay contact zone near the gingival fold, similar to stresses and strains. The average level of contact pressure and contact tangential stress is 3–4 and 7–8.9 times lower than the maximum values of the parameters respectively. The $\max\tau_{K}$ and $\Delta \tau_{K}$ are 7–9 and 15–19 times lower than $\max P_{K}$ and $\Delta P_{K}$. The study of the influence values of the friction coefficient on the biomechanical assembly deformation is required.

The obtained estimates give an idea of the qualitative patterns of the influence of the new restoration type and its materials on tooth deformation.

## 4. Discussion

### 4.1. Limitation Statement

This article is the result of preliminary research for a new type of restoration. The object of study has the following limitations:

- tooth root system in the model is discarded;
- interaction with gums is not taken into account;
- dental pulp is not modeled;
- enamel and dentin are deformed together;
- contact interaction is modeled as a complete adhesion of mating surfaces.

Material models have the following limitations:

- the behavior is described as isotropic elastic;
- materials shrinkage is not taken into account.

The main task of the work was to evaluate the effect of the material and geometric configuration of a new type of NCCL restoration on tooth deformation. The patterns of change in the deformation behavior of the restored tooth from the level of external load are also revealed. The accepted limitations are acceptable, but the geometric model and description of the materials behavior needs to be improved. The researchers face a number of tasks:

- the refinement of design schemes and finite element modeling of a biomechanical assembly;
- the analysis of the effect of the polymerization shrinkage of restorative composites on the total deformation of the tooth;
- the analysis of the influence of the cavity geometry for the prosthetic inlay in the NCCL;
- the analysis of the influence of materials for the restoration of an NCCL with different tooth configurations and cavities according to the prosthetic design;
- the analysis of the influence of loads from the antagonist tooth acting at an angle to the biomechanical system;
- the analysis of the influence of the nature of the tooth elements conjugation and the tooth-inlay system.

### 4.2. Materials

Researchers [50,51,52] note that one of the main criteria for the performance of NCCL restorations is adhesion between the restoration material and tooth elements. Battancs et al. concluded that the performance of the tooth-restoration system does not depend on the material. Thus, any material that already exists and is used in the treatment of an NCCL is suitable for prosthetics. Ichim et al. [3] come to the opposite conclusion and recommend the use of materials with a Young’s modulus of less than 1 GPa in restorations. The results of this work confirm that a material with a lower elastic compression modulus delivers a favorable strain behavior to the tooth-restoration system. At the same time, it should be noted that in the restoration of a harder material, a lower level of deformation is observed. Machado et al. come to the conclusion that non-straight ceramic inlays in the NCCL have less roughness, which favors subsequent periodontal treatment [53]. The authors of [53] also obtain information that composite restoration materials can reduce the stress level in the tooth-inlay system, but at the same time they have significant thermal shrinkage. Ceramic inlays and crowns have become widespread in the last decade [54,55]. Ceramic inlays make it possible to obtain good adhesion with the tooth elements, which is considered an important factor, especially in the prosthetics of an NCCL [55]. The maximum values of stress and strain intensity are observed near the contact between the lower part of the inlay and the tooth, which does not contradict the study data [3]. Additionally, there is a significant excision of the size of the tooth tissues, a significant number of defects, which probably does not only affect the increase in load, but also creates additional retention losses.

Today, there is a lot of research on the applicability of innovative biomaterials in dentistry. An interesting idea is to use oral-derived stem cells together with biomaterials or scaffold-free techniques to obtain strategic tools for regenerative and translational dentistry [56]. The authors [57] have developed a synthetic P26 peptide that demonstrates a remarkable dual mineralization potential to repair incipient enamel decay and mineralization defects localized in peripheral dentin below the dentin-enamel junction. The research in [58] indicated the prospect of using black phosphorene (BP) for pharmacological applications as scaffolds and prosthetic coverings. In our opinion, the combined use of innovative materials and new restorative inlay has great potential in the treatment of NCCLs and the prevention of their development. In the implementation of new clinical practices, it will be important to confirm them with the help of reliable computer models, which we are creating.

### 4.3. Influence of Taking into Account the Root System on the Tooth Deformation

Creating complete parameterized tooth geometry is a complex process. The modeling of teeth with a truncated root system [4,30,59] and without taking into account the root system [47,60,61] is often encountered in practice. Such limitations can have a significant impact on the numerical simulation results. Edge effects appear in the rootless model.

The root system of a tooth is often modeled with simplified geometry to pilot studies of new treatment methodologies [62,63]. The canonical geometry in the form of a single root is modeled [62,63]. The simulation of the simple geometry of two root canals is also encountered [35,64].

**Figure 12:**
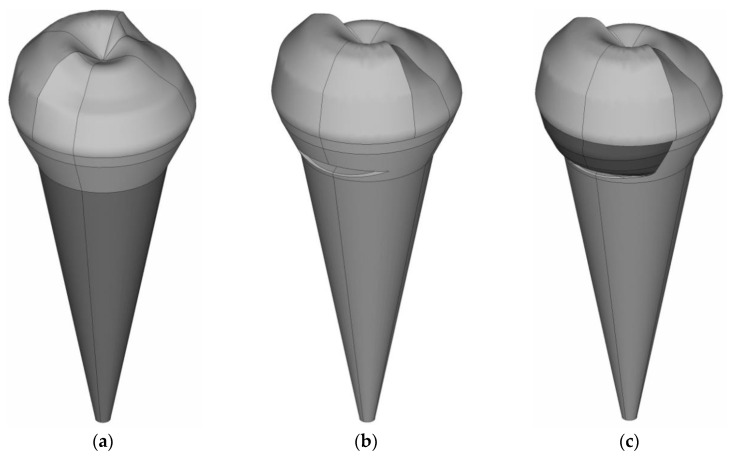
Models of teeth taking into account the root system simple geometry: (**a**) is without defect; (**b**) is with a defect; (**c**) is with the restoration new type.

Influence estimates of the tooth root system on the modeling results are of interest. It was decided to consider the tooth deformation (Figure 1 and Figure 3), taking into account the root system in order to clarify quantitative patterns. The tooth root system is modeled in a simplified setting (Figure 12).

The tooth root is modeled as a truncated cone. The boundary conditions are: the prohibition of the normal displacement of the side surfaces; the prohibition of all coordinates displacements of the root system lower part.

It has been established that taking into account the root system has little effect on the tooth deformation qualitative picture. A significant influence on the quantitative values of the deformation state parameters and the contact characteristics of the tooth-inlay system can be noted in this case.

**Figure 13:**
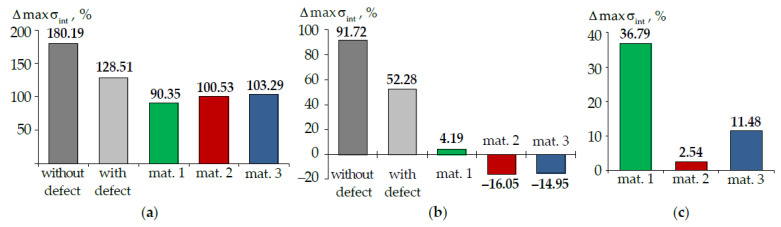
$\Delta \max\sigma_{\mathrm{int}}$: (**a**) is enamel; (**b**) is dentine; (**c**) is inlay; dark-grey is model without defect; light-grey is model with defect; green is model with material 1 inlay, red is model with material 2 inlay, blue is model with material 3 inlay.

**Figure 14:**
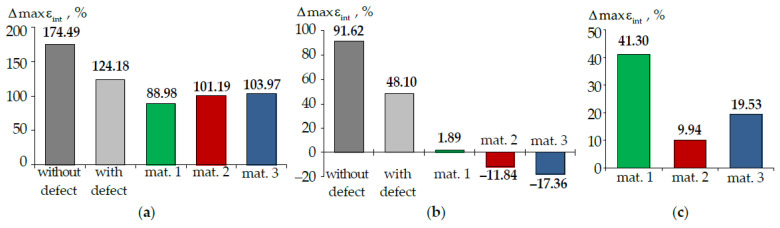
$\Delta \max\varepsilon_{\mathrm{int}}$: (**a**) is enamel; (**b**) is dentine; (**c**) is inlay; dark-grey is model without defect; light-grey is model with defect; green is model with material 1 inlay, red is model with material 2 inlay, blue is model with material 3 inlay.

**Figure 15:**
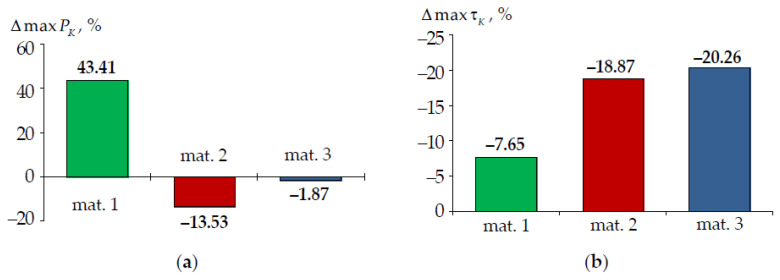
$\Delta \max P_{K}$ (**a**) and $\Delta \max\tau_{K}$ (**b**): green is model with material 1 inlay, red is model with material 2 inlay, blue is model with material 3 inlay.

The analysis of quantitative differences in the deformation behavior parameters of teeth with and without taking into account the root system will be performed according to the formula, where we take the model taking into account the root as reference values:

(1) $$\Delta A=\sum_{F=100}^{1000}\left[\left({A|}_{\mathrm{without}\;\mathrm{root}}-{A|}_{\mathrm{with}\;\mathrm{root}}\right)/{A|}_{\mathrm{with}\;\mathrm{root}}\cdot 100\right]/10\%,$$

where A is maximum parameters $\sigma_{\mathrm{int}}$ (Figure 13), $\varepsilon_{\mathrm{int}}$ (Figure 14), $P_{K}$ and $\tau_{K}$ (Figure 15). The difference percentage in deformation parameters slightly depends on the load. ΔA is the arithmetic mean of the deviation. The parameters were compared in terms of materials volumes in the crown area. The root volume was not taken into account when determining the parameters maxima.

Accounting for the root has the maximum effect on the behavior of the tooth without and with the defect. The maximum intensity of stresses and strains has a more pronounced localization in the defect area in this case.

The intensity of stresses and strain in the enamel is approximately two times lower in the tooth-inlay system when the root is taken into account. Accounting for the root system has an insignificant effect on the dentin deformation parameters (less than 20%).

The effect of taking into account the root on the contact parameters depends on the inlay material. For contact pressure: in material 1, there is a decrease in the parameter maximum level by approximately 40%; in materials 2 and 3, there is a slight increase in the parameter maximum level by 14 and 2%, respectively. The contact tangential stress in the model taking into account the root is higher.

The model without taking into account the root system gives only a qualitative idea of the tooth deformation. The refinement of models taking into account the root system close to real geometry is required. An analysis of the possibility of tooth root truncation to increase the computational procedures speed is also necessary.

This research direction is a priority for the scientific group. The rationalization of calculation schemes is necessary due to the wide scope of future research: influence analysis of the geometry cutout under inlay, inlay materials, occlusal load, the conjugation patterns tooth-inlay, etc.

### 4.4. Main Results

The distribution nature and the stress intensity level in a tooth with an NCCL are comparable to the results obtained by Jakupović et al. [9]. According to [3], the maximum stresses in the interface between the restoration and the tooth are observed near the edge of the lower surface of the inlay. The data obtained in this work show a similar result. An important result of the article is the investigation of a new technology for the restoration of an NCCL that allows the use of ceramic inlays that contribute to the creation of the required adhesion with the tooth elements. The use of a contact strain mechanism of the biomechanical system tooth restoration brings the nature of the strain closer to the real case. Many works on numerical analysis consider the strain of a tooth with the restoration of an NCCL within the framework of joint strain of the elements, which does not reflect all the features of the strain of a biomechanical assembly [3,9,32,33,34,35]. At the moment, a significant increase in stress in the dentine has been established, with a decrease in the stress level in the enamel at the inlay from the CEREC Blocs material. This effect can be avoided by the rational selection of the prosthetic inlay geometry and analyzing the influence of the tooth-restoration interface nature.

## 5. Conclusions

In general, the new restoration of the NCCL with the expansion of the cavity under the prosthetic inlay and the formation of the area of the gingival fold for the gum growth shows its viability. On the main volume of tooth materials, there are levels of strain parameters comparable to the tooth without taking into account the lesion. It is required to study the influence of the cavity geometry for the prosthetic construction and the veneer part of the inlay to eliminate local zones of the maximum level of stresses and strains in the biomechanical assembly. The problem of frictional contact interaction in the region of the tooth-inlay interface with the refinement of the finite element model should be extended.

It is established that the material of the Herculite XRV inlay (Kerr Corp, Orange, CA, USA) in this formulation of the problem delivers a favorable strain state to the biomechanical system. The nature and levels of the distribution of the stress values of the maximum and average level of the contact parameters of the interface zones and strain intensity are close to those of a tooth without taking into account the lesion. At the same time, the level of strain of the Herculite XRV inlay is comparable to the strains in the tooth enamel, which can adversely affect the service life of the structure.

The results obtained in this article reflect the qualitative patterns of tooth deformation behavior. Accounting for the root system and its rationalization is required for quantification.

## 6. Patents

A patent of the Russian Federation “Method of treating a wedge-shaped tooth lesion and a device for its implementation” No. 2 719 898, registration date 23 April 2020. The authors of the patent are Astashina Natalia, Petrachev Artem, Kazakov Sergei, Rogozhnikova Evgenia. The patent holder is Perm State Medical University named after E.A. Wagner.

## Figures and Tables

**Table 1:** Physical-mechanical properties of dental tissues.

Parameter	Enamel	Dentine
E, GPa	72.7	18.6
v	0.33	0.31

**Table 2:** Physical-mechanical properties of the prosthetic inlay materials.

Parameter	Material 1	Material 2	Material 3
E, GPa	45.0	9.5	14.1
v	0.3	0.24	0.24

**Table 3:** Comparison $\max\sigma_{\mathrm{int}}$ (%) in the biomechanical assembly elements of different models.

Model	Element	Model Accounting for Prosthetic Inlay		
		Material 1	Material 2	Material 3
Not taking into account the NCCL	Enamel	<by 16.00%	<by 11.86%	<by 12.93%
	Dentine	>by 154.62%	>by 15.17%	>by 38.83%
Taking into account the NCCL	Enamel	<by 10.65%	<by 6.24%	<by 7.38%
	Dentine	>by 23.39%	<by 44.19%	<by 32.72%

## Data Availability

Not applicable.
